# TB prevention strategies and unanswered questions for pregnant and postpartum women living with HIV: the need for improved evidence

**DOI:** 10.1002/jia2.25481

**Published:** 2020-03-23

**Authors:** Jyoti S Mathad, Sylvia M LaCourse, Amita Gupta, Jyoti Mathad, Sylvia LaCourse, Amita Gupta

**Affiliations:** ^1^ Department of Medicine and Obstetrics & Gynecology Center for Global Health Weill Cornell Medicine New York NY USA; ^2^ Department of Medicine Division of Allergy and Infectious Diseases University of Washington Seattle WA USA; ^3^ Department of Medicine and International Health Johns Hopkins University Baltimore MD USA

**Keywords:** tuberculosis, prevention, pregnant, HIV, isoniazid, postpartum

To end the tuberculosis (TB) epidemic, the World Health Organization (WHO) recommends improving access to testing and treatment of latent TB infection (LTBI) [1]. Despite recent epidemiologic studies demonstrating an approximately twofold increased risk of active TB in peripartum women [2], and poor outcomes associated with TB during pregnancy for mothers and their infants [3, 4], lack of data has prevented comprehensive inclusion of pregnant and postpartum women in guidelines and remains a critical gap in our efforts to address TB globally.

Because few TB studies include pregnant and lactating women, many questions remain, from TB epidemiology in pregnancy to optimal testing and treatment. In this viewpoint, we highlight knowledge gaps for TB prevention in pregnant and postpartum women living with HIV (PWLHIV) and opportunities to address them.

Of 10 million new TB diagnoses each year, 3 million occur in women, the majority of whom are of childbearing age [5]. Pregnancy status is not routinely collected and therefore not included in national or WHO global TB estimates, hampering our ability to understand the burden of disease in peripartum women.

A recent model estimates there are 150,000 pregnant and 50,000 postpartum women globally with incident TB each year [6]. In studies based on high‐burden settings, TB prevalence among pregnant women with HIV ranged from 0.8 to 11% [3]. Many of these studies are small, vary in design and quality of screening; therefore, the true burden of TB in pregnancy is not known. Furthermore, studies suggest pregnant women with TB may be asymptomatic, leading to further diagnostic delays.

A major challenge in TB prevention for pregnant women is identifying those at highest risk of progressing to active TB. WHO recommends the TB‐symptom screen to rule‐out active TB [1]. While early data from India suggested high negative predictive value in postpartum women [7], newer prospective data from multiple sub‐Sahara African countries suggests performance may be lower during pregnancy.

Once active TB is excluded, WHO recommends providing isoniazid preventive therapy (IPT) for people living with HIV, including pregnant women to prevent TB disease [1]. This recommendation has been called into question since the recently published TB‐APPRISE study found IPT initiation during pregnancy among PWLHIV increased the risk of adverse pregnancy outcomes [8]. (*see details in “Treatment” section below)*.

While some national programmes opt to first screen for LTBI, the optimal diagnostic test and timing remain unclear. The two most commonly used LTBI tests, tuberculin skin test (TST) and interferon gamma‐release assays (IGRA), demonstrate moderate agreement in non‐pregnant populations. Conversely, in studies among pregnant women in India and Kenya tested with both IGRA and TST, LTBI prevalence varied considerably (13‐49%) depending on the test (Figure **1**) [3, 9, 10].

**Figure 1:**
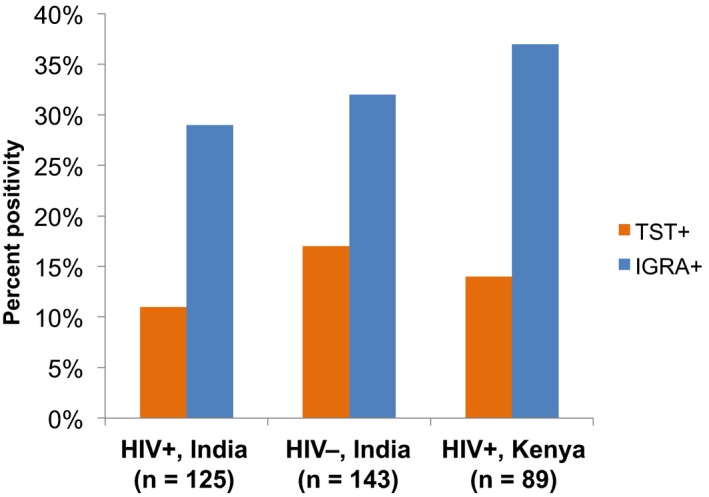
TST and IGRA perform differently in pregnant women with and without HIV.

Moreover longitudinal analyses suggest performance of these tests differ throughout pregnancy and the early postpartum period, with the number of positive results lowest during delivery and early postpartum, but increasing by 3 months postpartum [9, 10]. Pregnancy‐related decreases in CD4+ T cells that produce interferon‐gamma may impact testing results. Ongoing longitudinal cohorts in India and Kenya aim to evaluate whether immune changes of pregnancy impact host response to TB.

Among pregnant women in Ethiopia, the new IGRA, fourth generation QuantiFERON®‐TB GoldPlus, which measures interferon‐gamma production by CD4+ and CD8+ cells, had lower indeterminate results compared to the third generation Quantiferon Gold‐in‐tube^®^ IGRA test, with most pregnant women (89%) having adequate CD4+ and CD8+ interferon‐gamma responses with the fourth generation assay [11]. Regardless of LTBI test used, positive predictive value remains low in both pregnant and non‐pregnant populations, and new tests that diagnosis LTBI and better predict progression to active TB are urgently needed.

Isoniazid has been the cornerstone of TB prevention for over 50 years. Despite older studies from the United States reporting issues with hepatotoxicity and adherence during pregnancy, [12] more recent studies from sub‐Saharan Africa suggest adherence is high [13], even in early pregnancy, with minimal side effects reported.

IPT is widely accepted and recommended for use in all people living with HIV after excluding TB [1], with the recommendation for pregnant women based primarily on observational studies or secondary analyses of TB prevention trials of women who became pregnant on study [12, 14].

The first randomized control trial of IPT in pregnancy (TB‐APPRISE), demonstrates the importance of including pregnant and postpartum women in TB trials [8]. Gupta et al. reported PWLHIV on ART initiating IPT during pregnancy (in line with WHO recommendations), had a significantly increased risk of composite adverse pregnancy outcomes (e.g. stillbirth or spontaneous abortion, low birth weight, preterm delivery or congenital anomalies) compared to women initiating IPT 12 weeks postpartum. National programmes are now faced with the decision on whether to follow current WHO guidelines^1^ to administer IPT to PWLHIV in light of these data. In a recent evaluation of WHO TB preventative policy uptake in 38 high‐burden countries, approximately 1/3 did not make a specific recommendation regarding preventative therapy for PWLHIV.

Exclusion of pregnant women from drug trials has put more recently developed TB prevention regimens beyond their reach [15]. These newer regimens have fewer side effects and increased adherence compared to daily isoniazid in children and adults with and without HIV, with improved profiles largely attributed to shorter duration of therapy [16, 17]. Isoniazid (H) and rifapentine (P) is taken weekly for three months (3HP), or daily for one month (1HP). Shorter regimens could be ideal for pregnant women, allowing treatment completion before delivery, increasing adherence during routine antenatal care, and decreasing risk of postpartum progression to TB disease and transmission to young children. Safety and efficacy of these drugs in pregnant women require further investigation.

IMPAACT 2001 is a phase I/II trial of pharmacokinetics, tolerability and safety of 3HP in pregnant and postpartum women with and without HIV who have LTBI [18]. The study enrolled 50 pregnant women, including 20 with HIV, during their second or third trimester and followed them and their infants through 6 months postpartum. This innovative design allowed investigators to evaluate the effect of gestational age on drug metabolism for women and their infants. Results were presented at CROI 2020, but the study was not powered for safety as TB APPRISE was. A study of 1HP versus 3HP in pregnancy powered for safety and efficacy would ensure PWLHIV were offered data‐driven options for TB prevention.

High‐quality data are needed for safe and efficacious TB prevention in PWLHIV, yet widespread exclusion of pregnant women prohibit development of evidence‐based guidelines [15, 19]. TB APPRISE results highlight the potential harm of excluding pregnant women from trials. We can envision similar scenarios on the horizon, as we still have no recommendations on how to prevent or manage multidrug‐resistant TB in pregnancy.

Major questions that need to be urgently addressed include whether we should continue to provide TPT to all PWLHIV or employ a more targeted approach. If we do choose a targeted approach, how do we define women at high risk? In order to answer these questions, we need well‐designed studies, including population based‐analyses with clearly defined safety and effectiveness endpoints.

Finally, we must ensure benefits of new shorter regimens for TB prevention are extended to pregnant and postpartum women based on data. The innovative design of P2001, which accounted for changes in drug metabolism by gestational age, serves as an example for future studies of newer regimens, including 1HP. Small PK studies like P2001 are helpful, but we desperately need well‐powered studies to ascertain subtle safety effects from which we can perform risk‐benefit analyses. Pregnancy registries are also needed whereby first‐trimester exposure can be more readily assessed as we have done for HIV antiretroviral therapies in pregnancy. We have the tools we need; we just need the scientific and political will to include pregnant and postpartum women in our pursuit of ending the TB epidemic.

## Competing interests

The authors report no conflicts of interest.

## Authors’ contributions

AG initiated the concept to write this viewpoint. JSM developed the initial outline. JM and SML both contributed equally to the drafting of the initial draft of the manuscript. JM, SML and AG all reviewed, contributed to revision and approved the final manuscript.

## Funding

This work was supported by the National Institute of Allergy and Infectious Diseases (NIAID) (NIH/NIAID K23AI129854 and UM1AI068632 to JSM, NIH/NIAID K23AI120793 to SML, NIH/NIAID UM1AI069465 and NIH Eunice Kennedy Shriver National Institute of Child Health & Human Development (NICHD) R01HD081929 to AG. AG is also supported by JHU Center for AIDS Research 1P30AI094189, Ujala Foundation (PA, USA), and Wyncote Foundation (PA, USA).

### Disclaimer

The views represented are those of the authors’ alone and not their respective institutions or affiliated positions or funders.

## Authors' Information

Jyoti Mathad, MD, MSc, is an Assistant Professor in the Department of Medicine and the Department of Obstetrics and Gynecology at Weill Cornell Medicine. She is a mentored investigator with the IMPAACT (International Maternal Pediatric Adolescent AIDS Clinical Trials Network) TB Scientific Committee, co‐leads the Advocacy Arm of the International Union Against TB and Lung Disease Maternal Child Health Working Group and the Pregnancy Working Group of the TB Trials Consortium. Sylvia LaCourse MD, MPH, is an Assistant Professor in the Department of Medicine, Division of Allergy and Infectious Diseases, mentored junior investigator with the IMPAACT (International Maternal Pediatric Adolescent AIDS Clinical Trials Network) TB Scientific Committee, and International Union Against TB and Lung Disease Maternal Child Health Working Group Steering Committee, Scientific Arm Co‐Lead. Amita Gupta MD, MHS is a Professor of Medicine, Division of Infectious Diseases, and International Health at the Johns Hopkins University and serves on the TB Scientific Committees of IMPAACT, AIDS Clinical Trials Group (ACTG), International Epidemiologic Databases to Evaluate AIDS (IeDEA), and the Regional Prospective Observational Research in TB (RePORT) Consortia.
